# Physical activity and loneliness interrelations with retirement status, health, and personality factors: a theory-based study

**DOI:** 10.3389/fpsyg.2026.1844042

**Published:** 2026-07-15

**Authors:** Sonia Lippke, Petra Wagner, Volker Cihlar, Tiara Ratz, Yanping Duan

**Affiliations:** 1 Unit of Health Psychology & Behavioral Medicine, Constructor University Bremen gGmbH & Faculty of Health, Hamburg University of Applied Sciences (HAW Hamburg), Hamburg, Germany; 2 Institute of Exercise and Public Health, Faculty of Sport Science, Leipzig University, Leipzig, Germany; 3 Department of Aging, Federal Institute for Population Research, Wiesbaden, Germany; 4 AO Innovation Translation Center, AO Foundation, Davos, Switzerland; 5 Department of Sports and Health Sciences, Faculty of Arts and Social Sciences, Hong Kong Baptist University, Hong Kong, China

**Keywords:** continuity across the lifespan, extraversion, loneliness, mental health, neuroticism, partnership, physical exercise, physical health

## Abstract

**Objectives:**

Grounded in two theories, Evolutionary Theory of Loneliness/Feedback Model of Loneliness and Continuity Theory of Normal Ageing, primary objective of this study was to examine the longitudinal potentially bidirectional associations between loneliness and physical activity (PA) in later life. Three hypotheses were tested: (1) Loneliness and changes in PA are significantly associated over time when retirement status and partnership are controlled for; (2) Loneliness is correlated with PA, health, retirement, and personality factors; and (3) PA is correlated with loneliness, health, retirement, and personality factors.

**Design and methods:**

Data were drawn from a population-representative German cohort (*N* = 5,002; aged 54–70 years at baseline) participating in a longitudinal study across three waves: 2013 (T1), 2016 (T2), and 2019 (T3).

**Results:**

The proportion of physically active individuals increased over time (T1: 53.8%, T2: 58.6%, T3: 66.4%). Bivariate analyses supported Hypothesis 1, indicating a significant association between loneliness and PA over time. However, multivariate models revealed that health status was the strongest correlates of both loneliness and PA. After adjusting for health, partnership, and personality traits, PA is no longer significantly correlated with loneliness over time, partially supporting Hypothesis 2. Similarly, loneliness was not correlated with PA when controlling for past PA and health, offering partial support for Hypothesis 3.

**Conclusion:**

These findings underscore the central role of health in understanding patterns in older adults. Prior engagement emerged as strongest correlate of continued activity, while personality traits particularly lower neuroticism and higher extraversion and conscientiousness were associated with reduced loneliness. Interventions should therefore prioritize the early promotion of physically active lifestyles integrated with loneliness preventing behaviors and cognitions.

## 1 Introduction

Health-promoting behaviors, such as regular physical activity, play a critical role in promoting wellbeing in older adulthood. Yet these behaviors may be shaped by a complex interplay of psychological characteristics (e.g., personality), interpersonal circumstances (e.g., partner status), and broader environmental transitions (e.g., retirement), all of which can influence or interrelate with physical and mental health. *Health Psychology*, as an integrative field, seeks to explain how such psychological, biobehavioral, and contextual factors jointly contribute to salutogenesis (Mauro et al., 2025). Despite substantial advances, key questions remain regarding the pathways through which these influences operate, particularly in relation to loneliness in later life. Emerging evidence in populations approaching or entering retirement underscores both the progress made and the need for continued investigation. Advancing this work is essential for informing effective health psychology and public health strategies at individual, interpersonal, and societal levels, and this is the starting point for the current study. Before describing the empirical approach, the different aspects are outlined as well as the two theories underlying the research. Importantly, the present study is based on an *asymmetric measurement design*, in which physical activity is assessed repeatedly across multiple time points, whereas loneliness and health are measured at a single later time point (T3). Accordingly, the study focuses on how prior physical activity patterns are associated with loneliness at T3, while limiting conclusions regarding temporal dynamics or reciprocal processes.

*Loneliness* is increasingly recognized as a key determinant of mental health, particularly in the context of ageing and retirement (Salari et al., 2025). The *transition into retirement* represents a major life change that can affect loneliness (Field, 2025; Nyqvist et al., 2025; Puyané et al., 2025), social interaction and engagement in health-related behaviors, such as *physical activity* (PA) (Nazar et al., 2025). While PA is known to promote mental and physical health, its relationship with loneliness in later life remains complex (Gerber et al., 2025). Evidence suggests that PA may play a protective role in mitigating loneliness among *older adults* by fostering social connectedness, improving mood, and addressing age-specific challenges (Hong et al., 2024; Surkalim et al., 2024) although findings are mixed, particularly regarding long-term effects (Grillich et al., 2023; Hawkley et al., 2022; Shekelle et al., 2024). This underscores the need for a more nuanced and theoretically grounded understanding of how PA interacts with psychological and social processes in an ageing population-representative sample. In the present study, loneliness is assessed at T3 and is therefore conceptualized as a later-life outcome associated with prior PA patterns rather than a repeatedly observed process.

The *Evolutionary Theory of Loneliness* (ETL) (Cacioppo and Cacioppo, 2018) and the *Feedback Model of Loneliness* (Cacioppo and Hawkley, 2009) provide useful conceptual frameworks for interpreting these associations. ETL conceptualizes loneliness as an adaptive signal that motivates individuals to restore or maintain social connections, arising from interactions between dispositional factors (e.g., personality) and environmental conditions (e.g., work and retirement contexts). Consistent with this perspective, loneliness in later life has been associated with factors such as partnership status, socioeconomic conditions, health, and personality traits, including the Big Five dimensions (Buecker et al., 2020; Schutter et al., 2020). For instance, individuals with a high level of agreeableness tend to approach others more easily, exhibit positivity and openness, and are more likely to forgive after interpersonal conflicts. These traits can foster stronger and more resilient social connections, reducing feelings of loneliness (Berlingieri et al., 2024; Buecker et al., 2020; Dahlberg et al., 2022; Hajek and König, 2020).

At the same time, *health limitations*, such as chronic illness or reduced mobility may constrain opportunities for social participation and PA, thereby increasing vulnerability to loneliness (Hawkley et al., 2022; Luhmann and Hawkley, 2016). Although ETL emphasizes dynamic feedback processes between loneliness, health, and behavior, the present study cannot directly test such reciprocal mechanisms, as both loneliness and health are assessed only at T3. Instead, these constructs are treated as outcomes or contemporaneous correlates, and the theory is used as conceptual framework to inform the interpretation of observed associations as reflecting accumulated prior processes rather than ongoing feedback loops (Barnes et al., 2022; Berlingieri et al., 2024; Dahlberg et al., 2022; Hong et al., 2024; Luhmann et al., 2023; Naito et al., 2023; Qiao et al., 2022; Surkalim et al., 2024).

Contextual factors such as *retirement* further shape these associations. Retirement may either exacerbate or alleviate loneliness depending on changes in social roles and opportunities for engagement, including PA. For example, some studies suggest that PA increases following retirement, particularly when individuals reduce rather than fully cease employment (Van Scheppingen et al., 2014). However, others highlight increased vulnerability to loneliness, especially among men experiencing loss of work-related social networks (Puyané et al., 2025).

The *Continuity Theory of Normal Ageing* (Atchley, 1989) provides additional insight by proposing that individuals tend to maintain established behavioral patterns across the lifespan and to act as conceptual framework. This perspective is particularly relevant for PA, which is assessed longitudinally in the present study, allowing examination of behavioral stability over time. However, because loneliness and health are measured only at T3, continuity in these domains cannot be directly evaluated (Gerber et al., 2025). Despite the well-documented health benefits of PA, the mechanisms linking PA, loneliness, and psychological outcomes in older adulthood remain insufficiently understood.

Overall, existing research has yet to fully integrate theoretical models with longitudinal data to clarify how PA relates to loneliness in older adulthood. The present secondary data analysis seeks to address this gap by examining how prior PA trajectories are associated with loneliness at T3, while accounting for personality, health, retirement status, and partnership. Although theoretical frameworks such as ETL suggest dynamic and potentially reciprocal processes, the current data allow only limited inference regarding temporal directionality or causal mechanisms.

*Loneliness* is accordingly conceptualized primarily as:

- An *outcome*, as people who are more *physically active* feel more *physically healthy* and connected, and
- A correlate or potential *mediator*, as loneliness functions as an adaptive *signal for mental health*, motivating individuals to adopt/maintain, i.e., perform physical activity, and with that repair or seek out social relationships.

Therefore, loneliness was introduced at the later stage of the study, capturing its role after participants had experienced ageing-related challenges. This timing allows us to examine loneliness in relation to post-retirement circumstances rather than treating it as a baseline characteristic. The primary objective of this study was to examine the longitudinal potentially bidirectional associations interplay between loneliness and physical activity (PA) in later life. Accordingly, the research aimed to address the following *research questions*:

(1) Associations between PA trajectories and loneliness: How are levels and changes in PA across measurement points associated with loneliness assessed at T3 in older individuals, controlling for retirement and partnership status?
(2) Correlation with loneliness: To what extent is loneliness at T3 correlated with prior PA trajectories, and what role do health, retirement, and personality factors play in this association?
(3) Correlation with PA: To what extent is subsequent PA correlated with previous PA and loneliness at T3, when health, retirement, and personality factors are controlled for?

In line with the research questions, the *hypotheses* are as follows:

*Hypothesis 1*: PA levels and changes over time are significantly associated with loneliness at T3, when retirement status and partnership are statistically controlled for.

*Hypothesis 2*: Loneliness at T3 is significantly correlated with prior PA, as well as by health, retirement, and personality factors.

*Hypothesis 3*: Subsequent PA is significantly correlated with previous PA and loneliness at T3, as well as by health, retirement, and personality factors.

## 2 Methods

### 2.1 Design

Data originates from the study “Transitions and Old Age Potential” (TOP), which stems from a population-representative sample and is openly available. Details can be obtained from previous reports (Sackreuther et al., 2016). The study was conducted in accordance with the ethical principles outlined by the American Psychological Association (APA) and the 1964 Helsinki Declaration, including its subsequent amendments or comparable ethical standards. All study material, including interview questions and the procedures, underwent piloting to ensure clarity and reliability (Sackreuther et al., 2016).

### 2.2 Participants

**Figure 1:**
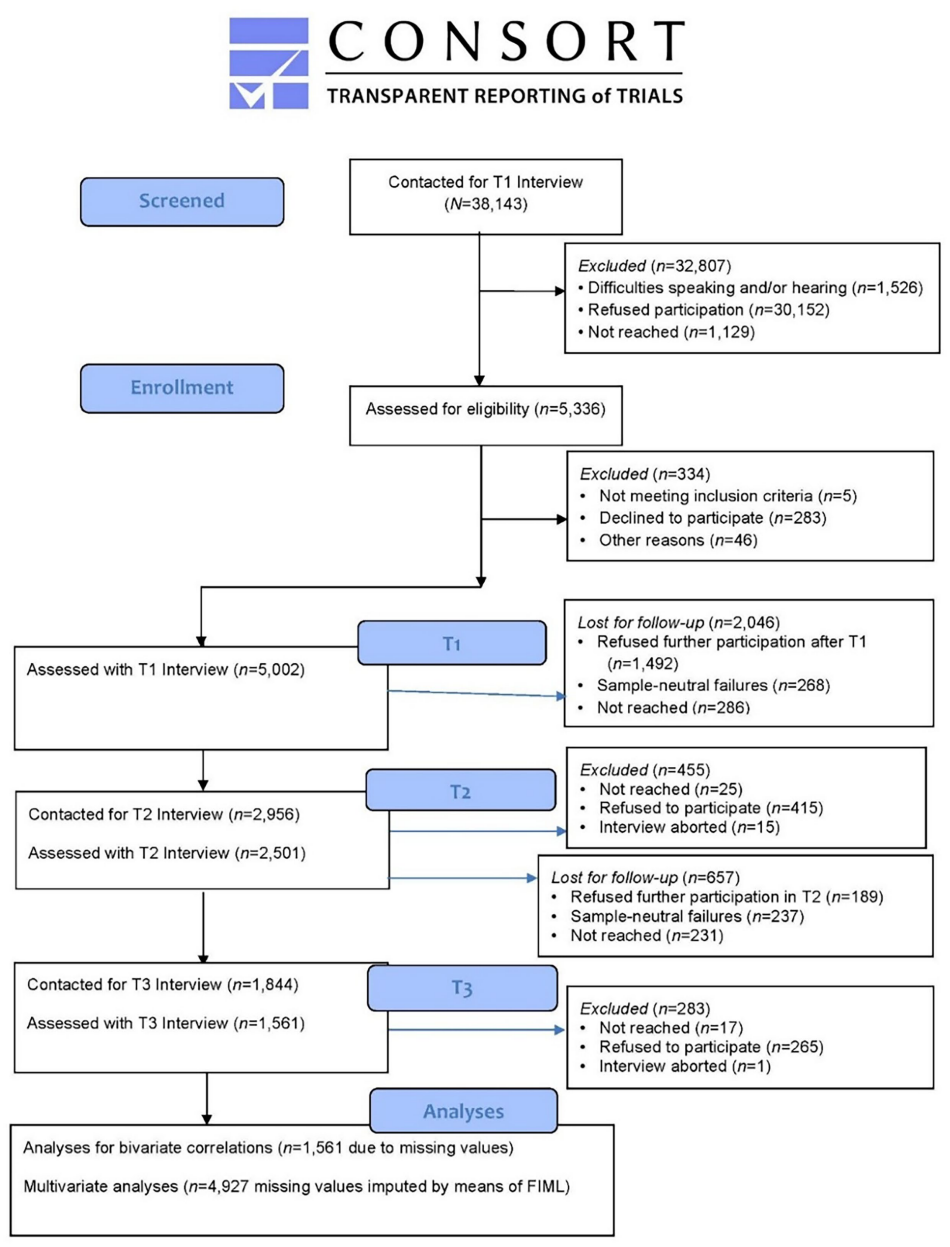
CONSORT flow chart with study participant flow over the three measurement points. We categorized study participants whom we ultimately could not reach as *sample-neutral failure* (Telephone number apparently did not work, no target person in the household, no private household) in contrary to not reached (Answering machine/No answer or Number busy/Promised to call back later but did not do so).

A total of *n* = 5,002 adults participated in the study at T1 (Figure 1). Over the course of 6 years, 1,561 individuals were interviewed during three waves of data collection: 2013 (T1), 2016 (T2), and 2019 (T3). This survey focused on individuals at the developmental phase to retirement and included participants aged 54 to 70 years at T1 who were either preparing for or adapting to retirement status. At the time of data collection, all participants resided in Germany.

#### 2.2.1 Procedure

Data were collected through computer-assisted telephone interviews (CATIs) and a standardized questionnaire. The overall coverage ratio at T1 was 13.1% (Figure 1), primarily due to individuals declining participation or not engaging in telephone interviews.

At the second measurement point (T2), a coverage ratio of 50% of the baseline (T1) sample was achieved. By the third measurement point (T3), a coverage ratio of 31.2% of the baseline sample was achieved. At T1, participants were between 54 and 70 years old (*M* = 62.08; SD = 4.71). Overall, *n* = 2,551 (51.0%) were retired and receiving a pension. About 55% were female (*n* = 2,738; *n* = 2,264 male). Most participants were living in a partnership (T1: 76.5%; T2: 76.4%; T3: 75.5%).

### 2.3 Measures

*Loneliness* was assessed at T3 using three items from the UCLA Loneliness Scale (Russell et al., 1978) in its short form (Hughes et al., 2004), based on four-point Likert scales. The loneliness index was calculated as the mean value of the three items (cf. Luhmann and Hawkley, 2016) with higher means indicating greater loneliness and median dichotomized. This loneliness index represents a dimensional variable that has been analyzed in previous work (Cihlar et al., 2022) using this dataset. Since this study is a secondary data analysis, we aimed to present the variable in a more distinct form. For selected subsequent analyses, we dichotomized the index to enable logistic regression, which offers a clear and interpretable framework for examining associations.

*PA* was determined by asking participants how often (<1, 1–2, 3–4, 5 + times per week) they were physically active for more than 30 min at T1, T2, and T3. This measure was used for the correlational and MANOVA analyses. Responses were dichotomized into insufficient PA (<1, 1–2) vs. sufficient PA (3–4, 5+) to facilitate interpretation and to align with public health thresholds in the Logistic Regression Analyses: The distinction between “insufficient” (<3 time per week) and “sufficient” (3+) physical activity is based on widely accepted public health recommendations, which emphasize that engaging in physical activity at least three times per week for 30 min is a minimal threshold for maintaining health benefits in older adults (Gerber et al., 2025).

*Subjective health status* was assessed using the validated SF12 v2 scale (Ware and Kosinski, 1996; Wirtz et al., 2018) with participants rating their *physical health* and *mental health* on a four-point scale (aggregated from 12 = poor to 73.5 = good health). Only T3 data of the SF12 were included in the analysis because we wanted to have more distal measures to loneliness instead of changes over time. *Retirement* was assessed at T1 by asking participants whether they received a pension, with responses transformed into a binary *pension/retirement* var*iable* (1 = yes, 2 = no). Personality traits, including *social agreeableness, openness to new experiences, neuroticism, extraversion,* and *conscientiousness,* were measured using the Big Five Dimensional Circumplex Questionnaire (Hofstee et al., 1992) with only T3 data analyzed.

The following sociodemographic variables at T1 were included: *Age* was calculated as the current year minus the year of birth. Binary *sex* was determined by the interviewer on basis of the name of the interviewee and a further question in case of uncertainties, because only male and female sex was differentiated, and no time was spent on exploring further genders by self-reports. Monthly household net equivalent *income* and *partnership status* were assessed by asking participants about their income and whether they had a partner. Educational attainment was measured using the International Standard Classification of Education (ISCED-97) classifications, based on the *highest level of general education* and *professional qualification,* and categorized into low, medium, and high education levels. An overview of all variables and their intercorrelations is presented in Table 1. For interval data, means (*M*) and standard deviations (*SD*) were calculated. For all ordinal or dichotomous variables (PA T1, PA T2, PA T3, Retirement, Partnership, Sex and ISCED) the median and mode were calculated (Table 1).

**Table 1:** Intercorrelations (Kendal Tau), *M* and SD (non-imputed data; *n* = 1,561).

	1	2	3	4	5	6	7	8	9	10	11	12	13	14	15	16	Mean/median/mode	*SD*
1. Loneliness (0 = never-3 = often)	1																0.80/0.667/1	0.62
2. PA T1 (days/w)	−0.053^*^	1															2.68/3.00/2	1.02
3. PA T2 (days/w)	−0.035	0.358^**^	1														2.76/3.00/3	1.00
4. PA T3 (days/w)	−0.088^**^	0.293^**^	0.320^**^	1													2.97/3.00/4	1.00
5. Agreeableness (low to high)	−0.091^**^	0.017	−0.012	0.003	1												3.51/3.67/4	0.47
6. Openness (low to high)	−0.105^**^	0.027	0.054^**^	0.052^*^	0.228^**^	1											3.23/3.33/3	0.55
7. Neuroticism (low to high)	0.175^**^	−0.015	−0.011	−0.038	0.113^**^	0.020	1										2.31/2.33/2.33	0.55
8. Extraversion (low to high)	−0.183^*^	0.069^**^	0.053^*^	0.053	0.421^**^	0.337^**^	-,009	1									3.39/3.33/4	0.51
9. Conscientiousness (low to high)	−0.154^**^	0.017	0.018	0.036	0.388^**^	0.272^**^	0.060^**^	0.357^**^	1								3.50/3.67/4	0.47
10. Physical Health (poor to good health)	−0.050^**^	0.051^**^	0.063^**^	0.114^**^	−0.077^**^	0.023	−0.067^**^	0.003	0.004	1							48.05	9.88
11. Mental Health (poor to good health)	−0.236^**^	0.042^*^	0.029	0.092^**^	0.006	0.090^**^	−0.205^**^	0.117^**^	0.106^**^	−0.075^**^	1						55.79	8.52
12. Retirement (1 = retired; 2 = working)	−0.001	−0.098^**^	−0.115^**^	0.010	−0.083^**^	−0.019	0.019	−0.071^**^	−0.026	0.127^**^	−0.006	1					1.52/2.00/2	0.50
13. Partnership (0 = yes; 1 = no)	0.089^**^	−0.012	−0.010	−0.017	0.032	−0.020	−0.065^**^	0.052^*^	−0.030	-,029	−0.020	−0.087^**^	1				1.20/1.00/1	0.40
14. Sex (0 = men; 1 = women)	−0.035	0.026	0.019	0.001	−0.233^**^	0.003	−0.051^*^	−0.161^**^	−0.018^**^	0.058^**^	0.022	−0.001	−0.197^**^	1			1.50/2.00/2	0.50
15. Age (younger to older)	<0.001	0.102^**^	0.115^**^	0.006	0.053^**^	0.020	−0.033	0.072^**^	0.010	−0.092^**^	0.036^*^	−0.608^**^	0.042	0.007	1		62.04/62.00/64	4.63
16. ISCED (low to high)	−0.022	0.068^**^	0.072^**^	0.058^**^	−0.188^**^	0.078^**^	−0.071^**^	−0.094	−0.024	0.108^**^	0.019	0.059^*^	−0.097^**^	0.224^**^	0.013	1	5.67/6/7	1.48
17. Income (Euro)	−0.077^**^	0.021	0.029	0.027	−0.094^**^	0.013	−0.045^*^	−0.045^*^	−0.005	0.113^**^	0.080^**^	0.023	−0.146^**^	0.108^**^	−0.009	0.351^**^	2,158.47/2,000/2,000	1,009.009

Missing data were not imputed with these binary analyses; PA, Physical Activity; T1, 2013; T2, 2016; T3, 2019; ISCED, International Standard Classification of Education. ^*^p < 0.05; ^**^p < 0.01.

### 2.4 Statistical methods

Analyses addressing the first research question and Hypothesis 1 were conducted using IBM SPSS v29. These included correlations, frequency analyses, ANOVA, and MANOVA. The methods were employed to examine associations between variables, analyze distributions of categorical variables, and test group differences, as well as the effects of loneliness and PA in different subgroups.

To address research questions/Hypotheses 2 and 3, logistic regressions for dichotomous dependent variables were used employing also SPSS because loneliness and physical activity were dichotomized to facilitate a clinically and practically meaningful distinction between higher-risk versus lower-risk groups, consistent with prior research and established cut-off approaches in the literature. This operationalization also supports interpretability in terms of the likelihood of experiencing loneliness or being physically inactive, which is particularly relevant for public health and intervention contexts. Given the binary specification of the outcomes, logistic regression was considered an appropriate analytical approach (Stoltzfus, 2011). Also testing Hypotheses 2 and 3, path analysis using Mplus version 8.6 were calculated. A maximum likelihood estimator with robust standard errors was used to account for potential non-normality in the data. Additionally, Monte Carlo integration was applied to model complex interactions. The dependent variables in the path analysis were loneliness and PA, both measured at T3 and defined as dichotomous indicators. Covariates included previous PA levels (T1, T2), health status (physical and mental health at T3), personality traits (Big Five at T3), retirement status (T1), partnership status (T1), age (T1), and sex (T1). Covariate selection was guided by a theoretically informed conceptual framework rather than a formal causal modeling approach. Specifically, included covariates were selected based on prior literature and their plausible roles as confounders of the relationship between loneliness and physical activity. These factors include sociodemographic characteristics (age, sex), past behavior (previous PA levels), health status (physical and mental health), and stable individual differences (personality traits), as well as contextual variables (retirement and partnership status). The selection strategy aimed to reduce potential confounding while avoiding overadjustment for variables that may lie on the hypothesized pathway. Given the observational nature of the data, the analyses are interpreted as estimating longitudinal associations rather than definitive causal effects. This approach reflects a balance between theoretical rigor and parsimony and is consistent with current recommendations for epidemiological and psychological research when formal causal diagrams are not specified.

Prior to conducting MANOVA, key assumptions were evaluated. Although PA was assessed as an ordinal frequency variable, it was treated as approximately continuous for these analyses, consistent with common practice for variables with multiple ordered categories. Inspection of distributions (skewness and kurtosis) and residuals indicated no substantial deviations from normality. Homogeneity of variance–covariance matrices was examined using Box’s *M* test, which did not indicate meaningful violations given the sample size and robustness of MANOVA to minor deviations. Equality of error variances was assessed using Levene’s test and was largely satisfied across groups. In addition, no evidence of problematic multicollinearity among dependent variables was observed. Taken together, these diagnostics suggest that the assumptions underlying MANOVA were sufficiently met to support the validity of the reported results.

To handle missing data, Full Information Maximum Likelihood (FIML) was utilized, allowing the inclusion of all available data without excluding cases with missing values. This methodological approach facilitated a comprehensive analysis of the correlations of PA and loneliness, taking into account individual and contextual factors.

Missing data primarily resulted from panel attrition across waves (T1–T3) and item-level nonresponse. PA exhibited missingness across all waves, whereas loneliness, health, and personality (all assessed at T3) were missing for participants not retained at follow-up. Descriptive and bivariate analyses (e.g., Table 1) were conducted on non-imputed observed data using pairwise or listwise deletion. Logistic regression models in SPSS employed listwise deletion, restricting analyses to complete cases. In contrast, path models in Mplus were estimated using Full Information Maximum Likelihood (FIML), which incorporates all available data under a missing-at-random assumption without prior imputation. Accordingly, results are based on analysis-specific samples: observed data (descriptives), complete cases (regressions), and FIML-based estimates (path models), which is considered when interpreting findings.

### 2.5 Dropout analysis

We examined whether study participants who completed all measurement points differed systematically from those who dropped out after T1 or T2 (Table 2). Systematic dropout was observed in 16 out of 19 tested variables. Differences were identified at T1 between participants who dropped out after the first measurement and those who either remained in the study for T2 or completed all measurement points. Specifically, differences were evident for variables including retirement status, partnership status, sex, agreeableness, extraversion, conscientiousness, ISCED education levels, income, physical and mental health (Table 2).

**Table 2:** Profile of study participants regarding socio-demographics, PA, personality factors, physical health, and mental health (all variables T1).

Construct with categories or range	T1 *M*, *n*	*SD*, %	T1–T2 *M*, *n*	*SD*, %	T1–T2–T3 *M*, *n*	*SD*, %	Test statistic
PA T1 times of 30 min/week	2.62	1.07	2.70	1.06	2.68	1.02	*F*(2,4,924) = 2.60
<1	426	17.4%	142	15.3%	210	13.5%	Chi^2^(6) = 16.81^*^
1–2	755	30.9%	278	29.9%	500	32.3%	
3–4	587	24.0%	227	24.4%	420	27.1%	
5+	678	27.7%	284	30.5%	420	27.1%	
Retirement							
Yes	1,281	51.2%	523	55.6%	747	47.9%	Chi^2^(2) = 14.32^**^
No	1,220	48.8%	417	44.4%	814	52.1%	
Partnership							
Yes	1866	75.2%	701	74.7%	1,244	79.7%	Chi^2^(2) = 13.14^**^
No	617	24.8%	238	25.3%	317	20.3%	
Sex							
Female	1,453	58.1%	507	53.9%	778	49.8%	Chi^2^(2) = 26.75^**^
Male	1,048	41.9%	433	46.1%	783	50.2%	
Age (54–70 years)	62.02	4.75	62.29	4.71	62.04	4.63	*F*(2,4,999) = 1.19
A (2–4)	3.55	0.47	3.56	0.46	3.46	0.50	*F*(2,4,949) = 18.83^**^
O (1.33–4)	3.26	0.59	3.28	0.58	3.29	0.55	*F*(2,4,961) = 1.20
N (1–4)	2.49	0.56	2.44	0.57	2.41	0.54	*F*(2,4,964) = 10.17
E (1.67–4)	3.44	0.52	3.45	0.52	3.39	0.51	*F*(2,4,957) = 5.48^**^
C (2–4)	3.52	0.48	3.54	0.47	3.47	0.47	*F*(2,4,948) = 6.82^**^
ISCED (1–8)	5.06	1.52	5.31	1.49	5.67	1.482	*F*(2,4,964) = 76.57^**^
Income (1–5,667)	2,496.61	1,292.43	2,596.97	1,285.99	2,900.48	1,451.53	*F*(2,4,999) = 144.10^**^
Physical Health (13.4–69.2)	48.94	10.57	49.05	10.44	50.81	9.37	*F*(2,4,880) = 17.22^**^
Mental Health (19.8–73.5)	53.46	10.06	54.09	9.71	54.51	8.71	*F*(2,4,880) = 5.873^**^
Loneliness T3	Not measured at T1 and T2				0.80	0.63	n.a.

Missing data are not imputed with these analyses; PA, Physical Activity; A, Agreeableness; O, Openness to Experience; N, Neuroticism; E, Extraversion; C, Conscientiousness; ISCED, Education Level. ^**^p < 0.01; ^*^p < 0.05.

## 3 Results

The results revealed an increasing percentage of participants categorized as physically active over time: with 53.8% active at T1, 58.6% at T2, and 66.4% at T3. Loneliness scores ranged from 0 (never) to 3 (often), with *M* = 0.80 (SD = 0.62), or after dichotomization 54.7% individuals were categorized with low levels of loneliness as they indicated to feel lonely „never “vs. 45.3% with high levels of loneliness, i.e., answering to feel lonely „rarely “to „often“. Loneliness was significantly correlated with PA at T1 (*r* = −0.05, *p* < 0.05) and T3 (*r* = −0.09, *p* < 0.05), but not at T2 (*r* = −0.04, *p* = 0.08; Table 1).

### 3.1 PA, loneliness, retirement, and partnership status (research question 1/Hypothesis 1)

**Figure 2:**
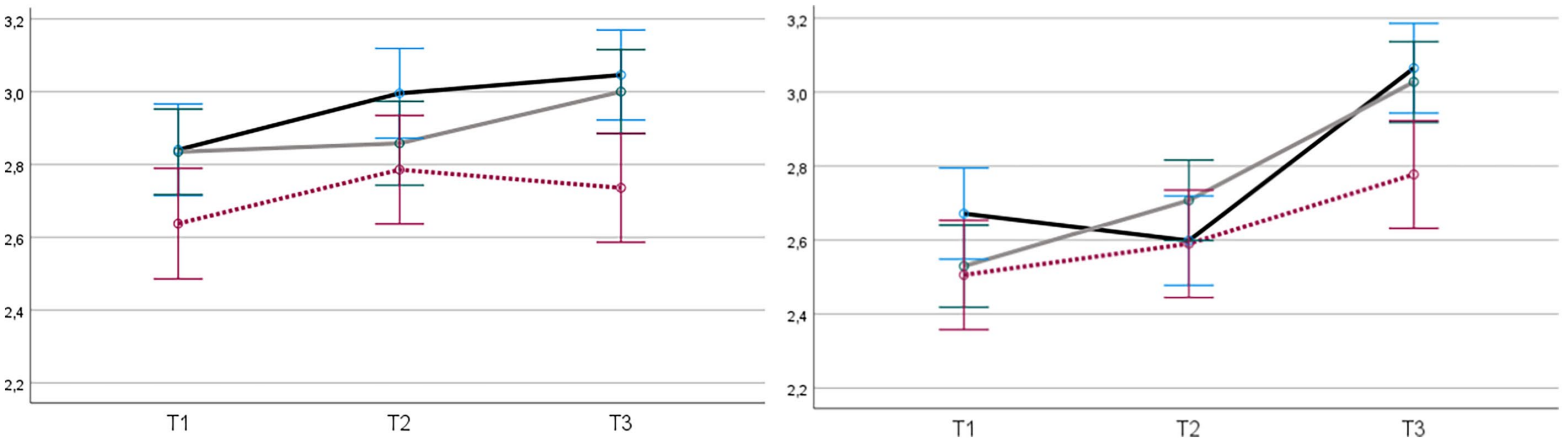
Physical activity (PA) levels of individuals who retired (left panel) and those who remained working (right panel) at three time points (T1, T2, and T3). Dotted line for high loneliness; grey line for medium loneliness and black line for low loneliness. *x*-axis: measurement points with *n* = 1,561 participating in all three measurement points, *y*-axis: self-reported PA (Ordinal measure). The analysis statistically controls partnership status.

A MANOVA examined the association between loneliness at T3 and changes in PA^1^ over time, controlling for retirement status and partnership. Loneliness was a significant factor, *F*(2, 1,515) = 7.65, *p* < 0.01, *η_p_*^2^ = 0.01, as was retirement, *F*(1, 1,516) = 12.28, *p* < 0.01, *η_p_*^2^ = 0.01. The interaction of previous PA and loneliness was also significant (*F*_Roy’s Largest Root_(2, 1,517) = 3.57, *p* = 0.03, *η_p_*^2^ = 0.01), as was the interaction between previous PA and retirement (*F*_Roy’s Lagest Root_(2, 1,516) = 12.29, *p* < 0.01, *η_p_*^2^ = 0.02). A triple interaction involving previous PA, loneliness, and retirement was likewise observed (*F*_Roy’s Lagest Root_(2, 1,517) = 4.48, *p* = 0.01, *η_p_*^2^ < 0.01; Figure 2).

Partnership did not emerge as a covariate (*F*_Roy’s Lagest Root_(2, 1,516) = 0.05, *p* = 0.95, *η_p_*^2^ < 0.01). These findings suggest that loneliness and retirement status were significantly associated with PA over time, with notable interactions among these variables, whereas partnership status had no detectable effect. Adults reporting high loneliness were less physically active at all measurement points. Additionally, individuals who were retired appeared more physically active than those who remained in the workforce (Figure 2). Individuals who were working at T1 showed larger increases in PA levels over time than individuals already retired. These results partially support Hypothesis 1, demonstrating significant associations between loneliness and changes in PA over time when controlled for retirement status and partnership – with retirement being a significant factor but partnership not.

### 3.2 Correlation with loneliness (research question 2/Hypothesis 2)

A logistic regression analysis was conducted to examine the interrelation between loneliness and physical activity (PA, binary measure) over time, and to explore the extent to which individual, social, and health-related factors were associated with these variables. The analysis included key determinants, such as health status, retirement, and personality traits (Table 3). The model fit was good as indicated by Chi^2^ (df = 13) = 229.35 (*p* < 0.01), −2 Log-Likelihood = 1,801.55; Cox & Snell *R*^2^ = 0.14. The independent variables explained 19% variance in loneliness (Nagelkerkes *R*^2^ = 0.193).

**Table 3:** Logistic regression results for correlation with loneliness at T3 (*n* = 1,561).

Variable (coding)	OR	95% C.I. for OR lower	95% C.I. for OR upper
PA T1 (insufficient PA vs. sufficient PA)	1.04	0.82	1.33
PA T2 (insufficient PA vs. sufficient PA)	1.15	0.90	1.47
Physical health T3 (poor to good health)	0.99	0.98	1.01
Mental health T3 (poor to good health)	0.95	0.93	0.96
Retirement T1 (retired vs. still working)	1.15	0.83	1.58
Partnership T1 (partner vs. no partner)	1.38	1.04	1.84
Agreeableness T1 (low to high)	0.79	0.58	1.06
Openness to experience T1 (low to high)	0.94	0.75	1.17
Neuroticism T1 (low to high)	2.09	1.67	2.62
Extraversion T1 (low to high)	0.62	0.47	0.82
Conscientiousness T1 (low to high)	0.62	0.47	0.82
Sex T1 (male vs. female)	0.85	0.67	1.08
Age T1 (younger to older)	1.03	0.99	1.06

PA, Physical Activity binary measure at T1 *n*_insufficient PA_ = 710 (45.5%), *n*_insufficient PA_ = 840 (53.8%), PA T2 3 years after T1 binary measure; *n*_insufficient PA_ = 637 (40.8%), *n*_insufficient PA_ = 914 (58.6%); loneliness (binary measure) as dependent variable at T3 6 years after T1 with *n*_iow-loneliness_ = 854 (54.7%), *n*_high loneliness_ = 706 (45.2%). Retirement T1 *n*_retired_ = 747 (47.9%), *n*_still working_ = 814 (52.1%). Partnership T1 *n*_partner_ = 1,244 (79.7%), *n*_nonpartnered_ = 317 (20.3%). Sex T1 *n*_male_ = 783 (50.2%), *n*_female_ = 778 (49.8%).

Results were validated using the imputed dataset and only one difference compared to the non-imputed dataset were found: With the imputed data set, also physical health was significantly interrelated with loneliness.

The analysis revealed that high levels of loneliness at T3 were not significantly correlated with PA at T1 or T2. Instead, loneliness was significantly interrelated with lower mental health, being single (i.e., no partnership), higher levels of neuroticism, and lower levels of extraversion and conscientiousness. This finding partially supports Hypothesis 2 in that loneliness is significantly correlated with mental health and personality factors, but -contrary to our expectation- no additional variance was explained by physical health, PA and retirement.

### 3.3 Correlation with PA (research question 3/Hypothesis 3)

The logistic regression model analyzing physical activity (PA, binary measure) at T3 as dependent variable indicated that sufficient levels of PA at T1 and T2 significantly explained PA at T3 (Table 4). Additionally, good self-rated physical and mental health at T3 were positively correlated with sufficient PA at T3. However, loneliness, personality factors, and the other tested independent variables did not show a significant relationship with PA at T3 (Table 4).

**Table 4:** Logistic regression results correlating with PA at T3 (*n* = 1,561).

Variable	OR	95% C.I. for OR lower	95% C.I. for OR upper
PA T1 (insufficient PA vs. sufficient PA)	0.43	0.33	0.56
PA T2 (insufficient PA vs. sufficient PA)	0.27	0.21	0.35
Loneliness T3 (continuous variable)	0.87	0.70	1.09
Physical health T3	1.04	1.03	1.05
Mental health T3	1.03	1.02	1.05
Retirement T1 (retired vs. still working)	1.25	0.88	1.80
Partnership T1 (partner vs. no partner)	0.98	0.72	1.33
Agreeableness T1	0.96	0.69	1.34
Openness to experience T1	1.00	0.78	1.28
Neuroticism T1	0.93	0.74	1.19
Extraversion T1	1.01	0.74	1.37
Conscientiousness T1	1.14	0.84	1.55
Sex T1 (male vs. female)	0.95	0.73	1.24
Age T1	0.99	0.96	1.03

PA, Physical activity binary measure at T1 *n*_insufficient PA_ = 710 (45.5%), *n*_insufficient PA_ = 840 (53.8%), PA T2 3 years after T1 binary measure; *n*_insufficient PA_ = 637 (40.8%), *n*_insufficient PA_ = 914 (58.6%); PA at T3 6 years after PA T1 binary measure; *n*_insufficient PA_ = 508 (32.5%), *n*_insufficient PA_ = 1,036 (66.4%). Retirement T1 *n*_retired_ = 747 (47.9%), *n*_still working_ = 814 (52.1%). Partnership T1 *n*_partner_ = 1,244 (79.7%), *n*_nonpartnered_ = 317 (20.3%). Sex T1 *n*_male_ = 783 (50.2%), *n*_female_ = 778 (49.8%).

Results were validated using the imputed dataset and only the effect of previous behavior was different compared to the imputed dataset: with the imputed data set it was found that behavior was likely to be maintained while in this non-imputed data set, individuals were more likely to adopt sufficient PA at T3 if they were insufficiently active at T1 and T2.

This model fitted the data well indicated by Chi^2^ (df = 14) = 291.45 (*p* < 0.01), −2 Log-Likelihood = 1,563.04; Cox & Snell *R*^2^ = 0.18. The independent variables explained 25% variance of PA at T3 (Nagelkerkes *R*^2^ = 0.25).

**Figure 3:**
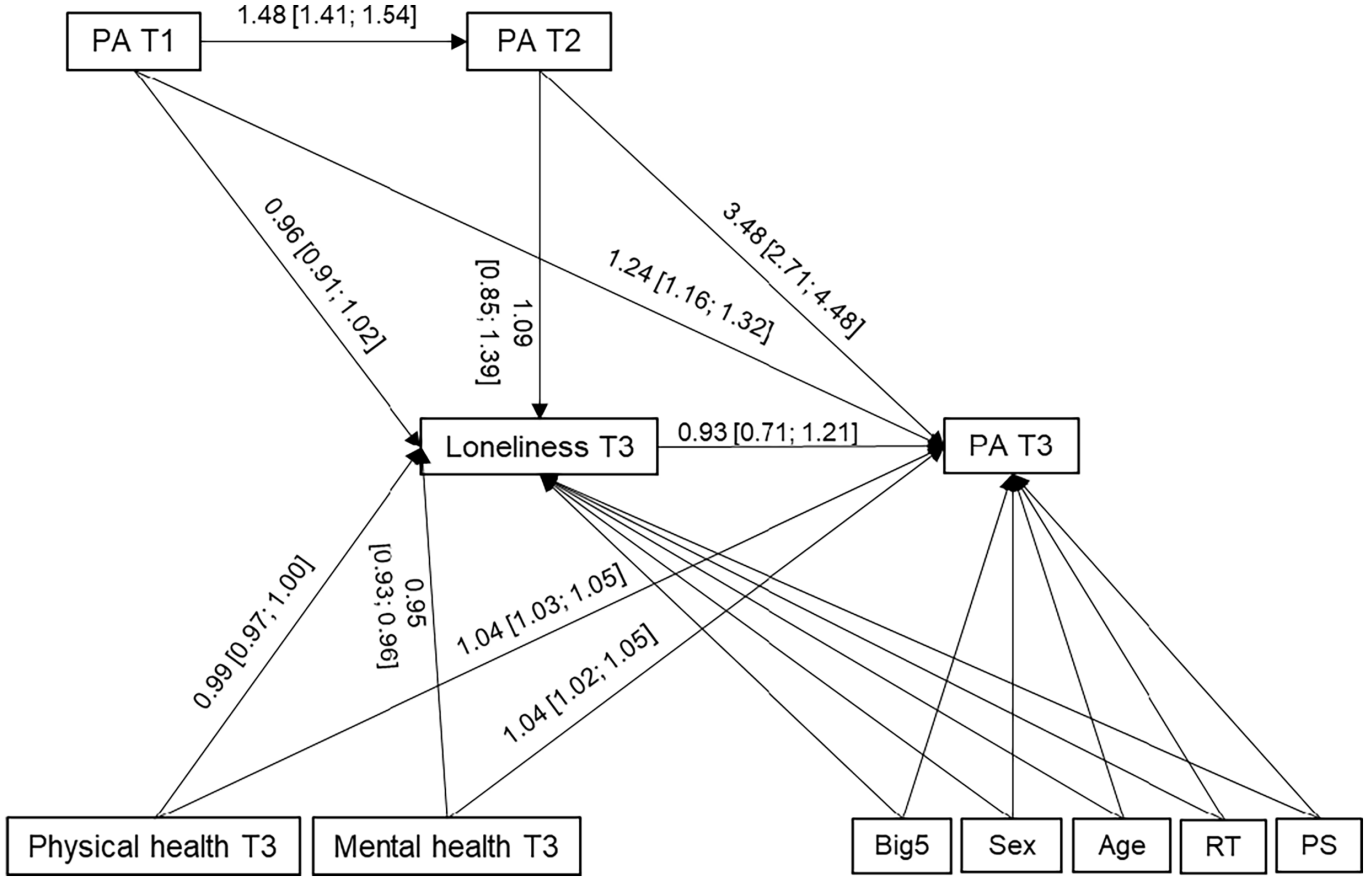
Path model of associations between loneliness (lower loneliness score as reference) and PA at three timepoints (insufficient levels of PA as reference). Odds Ratios and 95% Confidence Intervals; PA, Physical Activity (Ordinal measure), RT, Retirement, PS, Partnership; T2 3 years after T1 (PA T2 as ordinal measure); T3 6 years after T1 (PA T3 as ordinal measure); *n* = 4,927; Big5, Sex, Age, RT and PS at T1; Loneliness T3 (Continuous variable). Results were validated using the non-imputed dataset and no significant differences compared to the imputed dataset were found.

Bringing all aspects together, a confirmatory, à priori defined path model (Figure 3) was run, with a perfect model fit based on the following fit indices: root mean square error of approximation (RMSEA) < 0.01, comparative fit index (CFI) = 1.00, and Tucker-Lewis index (TLI) = 1.00.

This model further validated the logistic regression analysis that adults who engaged in PA at T1 were more likely to continue this behavior at T2 and T3, suggesting a rather stable pattern of PA over time. While PA and loneliness were not directly related when controlling for all other variables in the path model, physical and mental health appear to play an important role: participants with poorer self-rated health reported higher levels of loneliness. Conversely, adults with better mental and physical health were more likely to maintain sufficient PA levels at T3 (Figure 3).

The findings support Hypothesis 3 also only partially: PA is significantly correlated only with previous PA and health, but not by loneliness, retirement, or personality factors.

In conclusion, loneliness and PA demonstrated a correlation at the bivariate level. However, the primary explanatory factors of loneliness were health, partnership status, and personality traits such as neuroticism, extraversion, and conscientiousness. Conversely, the factors correlating with PA—when controlling for all other variables—were limited to past PA behavior and self-rated mental and physical health. These findings highlight the nuanced interplay between loneliness, health, and activity levels: While past behavior (PA) correlates with future behavior, and both PA and loneliness appear determined by health, PA and loneliness only share variance when the other factors are not taken into account. These findings and their meaning will be discussed in the following.

## 4 Discussion

In this prospective, theory-driven study we investigated loneliness and physical activity (PA) in a population-representative sample with 5,002 individuals aged 54 to 70 years. The conceptual frameworks of the *Evolutionary Theory of Loneliness* (ETL), the *Feedback Model of Loneliness* (Cacioppo and Hawkley, 2009) and the *Continuity Theory of Normal Ageing* (Atchley, 1989) suggested that behavior, loneliness and personality traits remain stable over time and that contextual factors such as retirement and physical activity shape experiences of loneliness (Field, 2025; Gerber et al., 2025; Nazar et al., 2025; Nyqvist et al., 2025; Puyané et al., 2025). To test this, participants were interviewed at the first measurement point and invited to be interviewed again at two follow-up measurement points. Although some dropout occurred, a substantial sample of 1,561 individuals provided data at all measurement points. Additionally, imputation methods were applied to prevent biases.

Higher levels of loneliness were associated -as suggested by the ETL- with higher neuroticism, but lower scores on other personality traits, specifically, agreeableness, openness, extraversion, and conscientiousness. Over time, an increasing number of participants achieved sufficient levels of physical activity. Lower levels of loneliness were associated with higher levels of physical activity at each time point, with this association being more pronounced among retired individuals. However, retirement did not correlate with loneliness; instead, loneliness variance was explained by health, partnership status, neuroticism, extraversion, and conscientiousness. Health was consistently associated with both loneliness as well as PA across all measurement points.

Thus, although past behavior (PA) is one of the strongest correlates with future behavior as hypothesized by the conceptual framework with its Continuity Theory. Its explanatory power often reflects the stability of underlying determinants rather than a direct causal link – which we also found in the current study. Both, physical activity and loneliness, constructs are influenced by more fundamental factors such as health status, retirement-related lifestyle changes, and stable personality traits (Schutter et al., 2020; Buecker et al., 2020; Hajek and König, 2020; Berlingieri et al., 2024; Dahlberg et al., 2022). These broader determinants shape individuals’ behavioral opportunities, motivational states, and social engagement patterns (Hawkley et al., 2022; Luhmann and Hawkley, 2016; Barnes et al., 2022; Berlingieri et al., 2024; Dahlberg et al., 2022; Hong et al., 2024; Luhmann et al., 2023; Naito et al., 2023; Qiao et al., 2022; Surkalim et al., 2022, 2024).

When these underlying factors are not statistically controlled for, PA and loneliness appear to share variance because both are distal outcomes of the same factors. For example, individuals with better physical health tend to be more physically active and simultaneously experience lower levels of loneliness due to greater functional capacity and social participation (Mauro et al., 2025). Similarly, personality traits such as extraversion or conscientiousness can encourage both higher activity levels and an extended social life, thereby reducing loneliness. Retirement status may also alter daily structure and social networks, impacting both activity patterns and cognitive representations of social connection (Field, 2025; Nyqvist et al., 2025; Puyané et al., 2025).

However, once these common determinants are explicitly accounted for in the analysis, the shared variance between PA and loneliness seem to diminish or disappear. This suggests that the detected bivariate association between PA and loneliness is largely artificial, stemming from their mutual dependence on these distal factors rather than from a proximal effect or direct relationship between the two. From an applied perspective, these findings suggest that interventions focusing exclusively on increasing physical activity may have limited effects on loneliness unless broader health, social, and personality-related factors are also addressed. Future intervention studies could explicitly test whether multifactorial approaches are more effective for improving physical activity and reducing loneliness than single-component interventions.

These findings are consistent with prior research (Gyasi et al., 2022; Hawkley et al., 2022; Schwartz et al., 2026), which has shown that loneliness and personality traits tend to remain relatively stable over time (Buecker et al., 2020). The results also align with the *Evolutionary Theory of Loneliness* (ETL) and the *Feedback Model of Loneliness* (Cacioppo and Hawkley, 2009), which highlight the role of personality traits (e.g., the Big Five) and contextual factors such as retirement and physical activity in shaping experiences of loneliness (Field, 2025; Gerber et al., 2025; Nazar et al., 2025; Nyqvist et al., 2025; Puyané et al., 2025). Moreover, the importance of supporting lonely individuals with health-related constraints, resonates with the ETL (Cacioppo and Cacioppo, 2018), which posits that psychological and health processes are embedded within social contexts (e.g., fewer social relationships with others). Notably, efforts to reduce the prevalence of loneliness (Barnes et al., 2022; Berlingieri et al., 2024; Grillich et al., 2023, Naito et al., 2023; Shekelle et al., 2024) and to promote PA levels by taking health constraints into account are equally essential (Cheng et al., 2025; Gerber et al., 2025; Hawkley et al., 2009; Pels and Kleinert, 2016; Surkalim et al., 2024; White et al., 2024).

Consistent with the *Continuity Theory of Normal Aging* (Atchley, 1989), PA appears to be a stable behavior: once initiated, it tends to be maintained over time, with individuals who were more active in the past showing greater increases in PA levels. The already discussed finding that the correlation between loneliness and PA vanished when health, personality, and demographic factors were controlled for, suggesting that PA may be less strongly associated with loneliness once health, personality, and demographic factors are taken into account (Pels and Kleinert, 2016). While PA fosters physical and mental health, and loneliness serves as an early indicator of health limitations (Arsenijevic and Groot, 2018; Barnes et al., 2022; Hajek and König, 2020), changing behavioral patterns might require additional motivators and resources, such as self-efficacy or structured planning.

Summarizing the results regarding the *research questions and hypotheses*: Although loneliness and PA were correlated at the bivariate level (Research Question 1/Hypothesis 1), health emerged as the primary driver or inhibitor of both outcomes. Loneliness appeared not to be correlated with PA longitudinally after controlling for key independent variables such as partnership status at baseline and concurrent health and concurrent personality factors (neuroticism, extraversion and conscientiousness; Research Question 2/Hypothesis 2). Similarly, PA was not correlated with loneliness once controlling for previous PA and health (Research Question 3/Hypothesis 3). Thus, health facilitates both engaging in PA and lower loneliness. Accordingly, future research may examine whether supporting ageing individuals in adopting health-promoting and physically active lifestyles also contributes to lower loneliness, particularly when health-related constraints are addressed. Furthermore, individuals may benefit from learning about potentials of personality characterized by low neuroticism, high extraversion, and high conscientiousness as early as possible. This could be interpreted and planned as a psychological strategy like cognitive efforts to address loneliness, i.e., the discrepancy between desired and experiences of social interactions (Luhmann et al., 2023).

### 4.1 Limitations and suggestions for future studies

As this study was correlational, future research should build on these findings by employing experimental designs to test interventions aimed at reducing loneliness and promoting PA jointly (Schwartz et al., 2026). Cross-lagged panel designs could further elucidate the temporal relationship between loneliness and PA over time (Dahlberg et al., 2022). In addition, future research would benefit from combining longitudinal designs with more fine-grained and multimethod assessments. While the present study allowed us to examine long-term associations between loneliness and physical activity, using more detailed measurement approaches may help to disentangle contextual, psychological, and health-related processes with greater precision.

Although this study did not have the goals to test how earlier levels of loneliness might have influenced subsequent PA behavior and loneliness perceptions, this might be of interest in the future and thus, loneliness should not only be assessed at the third time point but at all measurement points. Moreover, loneliness was assessed only at the third measurement point and using a global self-report measure. Future studies should assess loneliness repeatedly across measurement waves and employ differentiated instruments that distinguish between emotional and social loneliness, as well as between transient and chronic forms of loneliness. Such approaches may provide deeper insight into the temporal dynamics and heterogeneity of loneliness experiences over time. Additionally, further studies could examine the dynamic effects of retirement entry, maintenance, or changes in work hours aside retirement, as well as shifts in partnership status, health and other relevant factors (Schwartz et al., 2026).

Additionally, PA was assessed using a self-reported measure with limited granularity, which restricted the ability to capture within-person variation and situational context. Future research could benefit from more contextualized PA indicators, including information about activity type, intensity, and social context (e.g., solitary vs. group-based activity), which may be particularly relevant for understanding links between PA and loneliness. Furthermore, it was not captured whether PA was performed in a social context, which could function as a moderator by motivating individuals to adopt and maintain PA (McGowan et al., 2025). The large sample also means that the relatively small bivariate correlations may reach statistical significance, which should be interpreted with caution.

Furthermore, the study relied exclusively on subjective data, with some instruments lacking full validation. Future studies would benefit from multimethod approaches that combine self-reports with objective health indicators (e.g., clinical assessments, biomarker data) and objectively measured physical activity (e.g., accelerometry), which may reduce reporting bias and strengthen causal inference. Another concern is whether the findings can be generalized beyond this sample or replicated in other populations: the proportion of participants with a partner (75%) was much larger than singles, and much higher than in the general population (with 40%), suggesting a potential sampling bias. Future research should aim to overcome these limitations and explore whether similar patterns are observed in diverse cohorts and under varying circumstances, thereby validating and extending the findings of this study.

It is important to note that logistic regression was used to model dichotomous outcomes, and effect estimates are therefore expressed as odds ratios. Given that both loneliness and physical activity were relatively common in the sample, these odds ratios should not be interpreted as direct approximations of relative risks, as they may overstate the magnitude of associations. Accordingly, the reported estimates are interpreted as measures of association rather than risk. While alternative approaches such as modified Poisson regression with robust standard errors may yield more directly interpretable relative risk estimates (Gallis and Turner, 2019), the primary conclusions of this study are unlikely to depend on the specific modeling approach.

More broadly, the present analyses were designed to be explanatory rather than predictive. Covariates were therefore selected based on theoretical and empirical considerations, and results are interpreted in terms of longitudinal associations rather than predictive performance. In line with this approach, although the analyses were informed by a theoretically grounded selection of covariates, we did not employ a formal causal modeling framework such as Directed Acyclic Graphs (DAGs) to explicitly specify assumed relationships among variables. While such approaches can enhance transparency in identifying minimally sufficient adjustment sets and strengthen causal interpretations, the current study was primarily designed to examine longitudinal associations rather than to draw definitive causal inferences. Accordingly, findings should be interpreted with appropriate caution.

Future research may build on the present work by replicating the analyses with continuous dependent variables, incorporating alternative modeling strategies (e.g., modified Poisson regression), formal causal frameworks (e.g., DAG-based approaches), and validation-oriented methods, thereby further strengthening the robustness, transparency, and interpretability of observed relationships between loneliness and physical activity.

Furthermore, although post-stratification weighting could be used to align the partnership distribution with population benchmarks, such procedures do not address sampling bias in a causal sense but rather recalibrate population-level estimates; given the health psychology focus of this special issue and our emphasis on associative mechanisms and individual-level processes, as well as the limited use of weighting in comparable psychological research, we therefore did not apply partnership-based weights in the present analyses. Taken together, these findings highlight the need for future longitudinal research that integrates fine-grained psychological measures, contextualized behavioral indicators, and objective health data to better understand the complex interdependencies between loneliness and physical activity in later life.

## 5 Conclusion

In this population-representative sample of adults aged 54–70 years, individuals reporting higher levels of loneliness tended to engage in physical activity (PA) at the bivariate level. However, once health status, prior physical activity, partnership status, and personality traits were taken into account, physical activity did not emerge as a longitudinal correlation with loneliness. Loneliness was more strongly associated with poorer self-rated health, being without a partner, higher neuroticism, and lower extraversion and conscientiousness. Given the critical role of PA in maintaining or improving health in older adults, these findings underscore the potential relevance of psychological and social factors for understanding co-occurring patterns of physical activity and loneliness. Future studies should test whether interventions that jointly address social, psychological, and health-related factors are more effective in promoting sustained physical activity and reducing loneliness than approaches targeting behavior alone (McGowan et al., 2025).

These patterns underscore the complex interplay of psychological, interpersonal, and environmental processes that shape health behaviors in later life. As normative retirement transitions within this cohort may coincide with both loneliness and changes in activity routines, understanding these interdependencies may inform the development and testing of health psychology-informed interventions (Schwartz et al., 2026; Mauro et al., 2025). Continued efforts are needed to support resources to adopt and maintain a healthy lifestyle and with that to prevent loneliness, and to design according to health psychological approaches that support ageing populations in these life transition phases, building on the current study and its results.

Accordingly, the implications of these results should be interpreted with appropriate caution. Given the observational design and the specific age range, health profile, and partnership composition of the sample, the findings are most applicable to adults transitioning from late midlife into early older age, particularly in contexts characterized by relatively stable social structures. While physical activity remains a key determinant of health in later life, interventions aiming to reduce loneliness in comparable populations are unlikely to be effective if they focus solely on increasing PA without also addressing broader psychological, social, and health-related factors. Retirement and related life transitions may represent important contexts in which these interdependencies become salient; however, the meaning and consequences of such transitions are likely to vary across social and cultural settings.

Overall, the present study underscores the importance of situating PA and loneliness within a broader constellation of health, personality, and social resources. Future research should test whether multifaceted, conceptual framework and theory informed interventions that are tailored to specific population contexts -and that explicitly take sampling and contextual boundaries into account are more effective than single component approaches in supporting healthy and socially connected ageing.

## Data Availability

Publicly available datasets were analyzed in this study. This data can be found here: the dataset is openly available as a public user file via https://search.gesis.org/research_data/ZA6597.

## Footnotes

1 Ordinal measure of PA (PA T1 times of 30 min/ week: <1; 1–2; 3–4; 5+).
